# Non-Intrusive Pipeline Flow Detection Based on Distributed Fiber Turbulent Vibration Sensing

**DOI:** 10.3390/s22114044

**Published:** 2022-05-26

**Authors:** Ying Shang, Chen Wang, Yongkang Zhang, Wenan Zhao, Jiasheng Ni, Gangding Peng

**Affiliations:** 1 Laser Institute, Qilu University of Technology (Shandong Academy of Sciences), Jinan 250014, China; shangying@sdlaser.cn (Y.S.); zhangyongkang@jiqt.org (Y.Z.); wenan.zhao@sdlaser.cn (W.Z.); njsh@qlu.edu.cn (J.N.); 2 Jinan Institute of Quantum Technology, Jinan 250101, China; 3 School of Electrical Engineering and Telecommunications, University of New South Wales, Sydney, NSW 2052, Australia; g.peng@unsw.edu.au

**Keywords:** distributed acoustic sensor, flow monitoring, turbulent vibration, non-intrusive detection

## Abstract

We demonstrate a non-intrusive dynamic monitoring method of oil well flow based on distributed optical fiber acoustic sensing (DAS) technology and the turbulent vibration. The quantitative measurement of the flow rate is theoretically acquired though the amplitude of the demodulated phase changes from DAS based on the flow impact in the tube on the pipe wall. The experimental results show that the relationships between the flow rate and the demodulated phase changes, in both a whole frequency region and in a sensitive-response frequency region, fit the quadratic equation well, with a max R^2^ of 0.997, which is consistent with the theoretical simulation results. The detectable flow rate is from 0.73 m^3^/h to 2.48 m^3^/h. The experiments verify the feasibility of DAS system flow monitoring and provide technical support for the practical application of the downhole flow measurement.

## 1. Introduction

In the development and production of oilfields, tracking and monitoring small downhole flow are carried out to obtain the production performance of each production layer of the oil well. However, for now, instruments such as mechanical and electronic flowmeters, which have been widely used in the industry, still have risks of a low reliability, high cost and downhole intervention, and are difficult to use in harsh downhole environments [1]. The optical fiber flowmeter has the advantages of a large dynamic range, high response sensitivity and anti-electromagnetic interference, which makes it increasingly widely used in the field of flow monitoring [2,3].

With continuous exploration, the fiber optical flowmeter mainly uses fiber Bragg grating (FBG) as the core device. Based on multimode optical fiber coupling, Chen et al. developed an FBG single point flow sensor for the first time, with a measurement sensitivity of up to 0.35 m/s [4]. Schen et al. used the quadrant position sensitive detector to receive the spot position emitted by the optical fiber probe, which can realize a gas flow measurement of 2 L/min [5]. Zhao et al. designed a micro-probe FBG flowmeter via a hollow cylinder to measure the fluid impact, which had a flow rate of 0~22.5 m^3^/h with a resolution of 0.81 m^3^/h [6]. Lv et al. designed a target flowmeter that combined a capillary tube with double FBG, which could measure the flow and temperature at the same time. This flowmeter achieved a measurement deviation of less than 0.25 m^3^/h in the 5~16 m^3^/h measurement range [7].

However, the above optical fiber flowmeters have to place the sensor inside the pipe, which will hinder the fluid in the pipe. Additionally, there will be a liquid residue on the sensor, which affects the measurement accuracy. Therefore, the study of a non-intrusive optical fiber sensing method is of great significance for pipeline flow monitoring, and the use of a turbulent vibration of fluid in the pipeline for flow detection has become a research hotspot in recent years [8,9,10,11]. Shang et al. used the FBGs combined with the interference demodulation method to achieve a non-intrusive flow measurement, which had a measurement range of 5.7–46.5 m^3^/h [12]. Ni et al. designed a new type of optical fiber flowmeter based on the distributed feedback fiber laser (DFB-FL), which is placed on the outer wall of the pipe, to measure the accurate small flow from 0.71 m^3^/h to 2.35 m^3^/h [13]. Although the above sensors have an enhanced sensitivity in structure, they are still a point sensor, which can only monitor at a certain position on the pipeline. Distributed fiber acoustic sensing (DAS) technology can quantitatively detect the external physical quantities along the whole fiber, and the measurement accuracy has been greatly improved. At present, it plays a great role in the field of pipeline leakage and other long-distance monitoring [14,15,16,17,18].

In this paper, a new type of distributed optical fiber flow sensing system is designed, and the pipeline fluid simulation model is established based on the principle of turbulent vibration. The relationship between the flow rate and demodulated phase changes is given, and the experimental result shows a good quadratic fitting between the flow rate and the demodulated phase changes in both a whole frequency region and in a sensitive-response frequency region, which verifies the stability and accuracy of the DAS system under a small flow.

## 2. Theoretical Analysis

### 2.1. Principle of Flow Measurement Based on Turbulent Vibration

**Figure 1:**
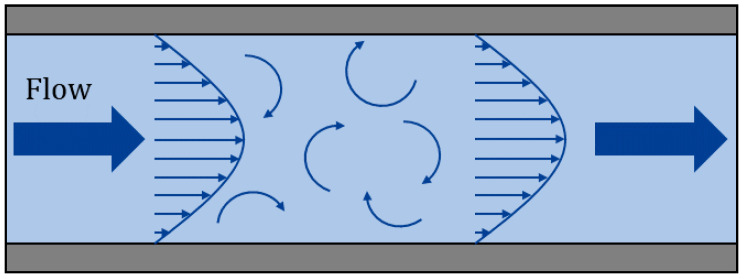
Turbulent vibration in pipe.

In hydrodynamics, in addition to describing the motion of the fluid, it is necessary to consider the force acting on the fluid. As shown in Figure 1, the Euler method is chosen to analyze the motion of the fluid turbulent vibration.

In general, the flowing fluid energy can be divided into three types: potential energy, internal energy and kinetic energy. The differential form can be expressed as:

(1) $$\rho \frac{d}{dt}\left(U+\frac{V^{2}}{2}\right)=\rho F\cdot v+div\left(P\cdot v\right)+div\left(kgradT\right)+\rho q,$$

In this equation, the left side represents the corresponding rate of change of the fluid energy per unit mass, and the right side indicates the work done by the mass force, surface force, heat and radiant heat on the unit mass fluid per unit time.

When the fluid in the pipe is in a state of motion, there is a certain impact on the pipe wall, and the fluid will transfer energy while impacting the pipe wall, and most of the kinetic energy will be transformed into pressure to act on the pipe wall. This causes the pipe wall to vibrate at a certain frequency [19].

The velocity of the fluid passing through the cross-section of the horizontal tube can be expressed as the following two equations:

(2) $$u=\bar{u}+u^{\prime },$$

(3) $$v=\bar{v}+v^{\prime },$$

where *u* represents the velocity parallel to the axial direction of the pipeline, $\bar{u}$ represents the average velocity in that direction, and *u*′ represents the instantaneous velocity in that direction; *v* represents the velocity parallel to the radial direction of the pipe, $\bar{v}$ represents the average velocity in that direction, and *v*′ represents the instantaneous velocity in that direction.

For a circular pipe, the relationship between the pressure *p* and the velocity of the internal fluid acting on the pipe wall follows:

(4) $$p\propto \bar{u^{\prime }v^{\prime }},$$

The product of the instantaneous velocity of the fluid in different directions is not zero, which is *u*′*v*′ ≠ 0

The pipe filled with liquid is simplified to a one-dimensional beam, combined with the theory of engineering mechanics:

(5) $$\frac{d^{2}M}{dx^{2}}=\frac{dV}{dx}=p^{\prime }\left(x\right),$$

where *M* is the bending moment, *V* is the shear force, and *x* is the axial displacement.

When the beam is subjected to bending, one side of the beam is stretched and the other side is compressed, there is a bending equation:

(6) $$M=EI\frac{d^{2}y}{dx^{2}},$$

where *E* is the elastic modulus, *I* is the moment of inertia, and *y* is the radial displacement.

Pressure fluctuations can be obtained by bringing Equation (6) into Equation (5):

(7) $$p^{\prime }\left(x\right)=\frac{d^{2}M}{dx^{2}}=EI\frac{d^{4}y}{dx^{4}},$$

The acceleration of the radial displacement of the pipe can be solved according to the differential equation of motion of the transverse vibration of the beam:

(8) $$\frac{\partial^{2}y}{\partial t^{2}}=\frac{\partial^{2}M}{\partial x^{2}}=-\frac{g}{A\gamma }EI\frac{\partial^{4}y}{\partial x^{4}}=-CEI\frac{\partial^{4}y}{\partial x^{4}}=-Cp^{\prime }\left(x\right),$$

where *A* is the cross-sectional area of the beam, *γ* is the specific gravity of the beam, and *g* is the gravitational acceleration.

It can be seen from Equation (8) that the acceleration of the pipe wall is proportional to the pressure fluctuation in the fluid, and the ratio of the instantaneous velocity to the average flow is constant. We define this value as the flow intensity, which is a measure of the amplitude of the fluid disturbance: $\sqrt{\bar{m}}/\bar{u}$ is the flow intensity. We also define *m = u*′^2^, and $\sqrt{\bar{m}}$ is the root mean square of the instantaneous velocity *u*′. Combined with the velocity formula of the cross-section of the horizontal tube given above, we can get:

(9) $$\frac{\sqrt{\bar{m}}}{\bar{u}}=\frac{\bar{m}}{{\bar{u}}^{2}}=\frac{\frac{1}{N}\sum_{i=1}^{n}{\left[u_{i}\left(t\right)-\bar{u}\right]}^{2}}{{\bar{u}}^{2}}=C,$$

Equation (9) can also be written as:

(10) $$\frac{1}{N-1}\sum_{i=1}^{n}{\left[u_{i}\left(t\right)-\bar{u}\right]}^{2}=\frac{NC}{N-1}{\bar{u}}^{2}=k{\bar{u}}^{2},$$

so that:

(11) $$p\propto k{\bar{u}}^{2}.$$

Through the above theoretical analysis, there is a significant positive proportional relationship between the standard variance of pipeline fluid vibration and its corresponding average flow rate, and there is also a significant quantitative relationship between the standard variance of vibration acceleration and $\bar{u}$. When we get the standard variance of vibration energy in the flow test, we can calculate the average velocity.

### 2.2. Mechanism of Optical Fiber Pressure-Phase Modulation

**Figure 2:**
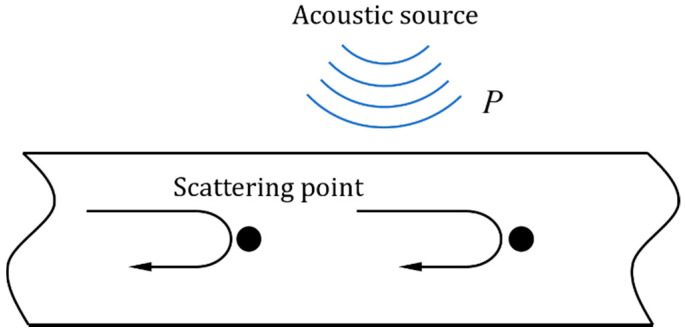
Mechanism of the pressure-phase modulation.

When the external acoustic signal acts on the fiber, it can be regarded as the external pressure on the optical fiber, as shown in Figure 2, which changes the fiber length, refractive index, and other parameters. The change of these optical fiber parameters will change the phase of the optical wave transmitted inside the fiber, and finally be demodulated [20].

When the external acoustic pressure acts on the optical fiber, the phase change of the light can be expressed as follows [21]:

(12) $$\Delta \varphi =\frac{4\pi n_{f}l}{\lambda }\varepsilon (1-P_{e}),$$

where λ represents the light wavelength, *l* represents the sensing fiber length, *n_f_* represents the refractive index, *P_e_* represents the effective photoelastic coefficient, and *ε* represents the axial strain of the fiber.

The pipe can be seen as a cylinder [22], so that the strain on the outer wall of the pipe caused by its internal pressure *p* is:

(13) $$\varepsilon =\frac{r_{1}^{2}}{E(r_{2}^{2}-r_{1}^{2})}p,$$

where *E* represents Young’s modulus, *r*_1_ represents the inner diameter of the pipe, and *r*_2_ represents the outer diameter of the pipe. Thus, the following equation is obtained:

(14) $$\Delta \varphi =\frac{4\pi n_{f}l}{\lambda }(1-P_{e})\frac{r_{1}^{2}}{E(r_{2}^{2}-r_{1}^{2})}p,$$

By combining Equation (11) with Equation (14), we can get:

(15) $$\Delta \varphi \propto k{\bar{u}}^{2},$$

From Equation (15), we finally get that the phase difference Δ*φ* is proportional to the square of the average velocity $\bar{u}$, and the flow rate value can be obtained by calculating the phase changes.

### 2.3. Spatial Differential Interference Detection

**Figure 3:**
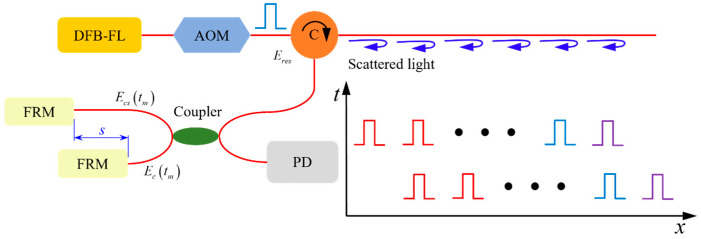
Spatial difference interference principle of the DAS system.

In this paper, a spatial differential interference DAS system is designed based on the structure of the Michelson interferometer, as shown in Figure 3.

Each scattering point along the optical fiber is regarded as a discrete equivalent mirror, and the phase difference is obtained by making a spatial difference between the two points. When the external pressure acts on the optical fiber, the phase at this position will change accordingly. Furthermore, the phase difference at the pressure position can be obtained. After that, the acoustic signal can be restored by demodulating the phase through the phase demodulation technology.

Each scattering point along the optical fiber is regarded as a discrete equivalent mirror, and the light intensity received at the detector can be expressed as:

(16) $$I_{z}\left(t_{n}\right)=A+B\cos\left(\varphi_{m,m-s}\right),$$

where *φ_m,m−s_* is the phase difference of the backward Rayleigh scattered light at the *m* and *m-s* equivalent mirrors.

When the external pressure acts on the optical fiber, the phase at this position will change with it, which can be expressed as:

(17) $$\varphi_{m,m-s}=\varphi_{m-s}-\varphi_{m}+\Delta \varphi ,$$

where *φ_m−s_* − *φ_m_* is the initial phase difference and is generally a fixed value; through the phase demodulation technology to demodulate *φ_m,m−s_*, the acoustic signal can be restored.

**Figure 4:**
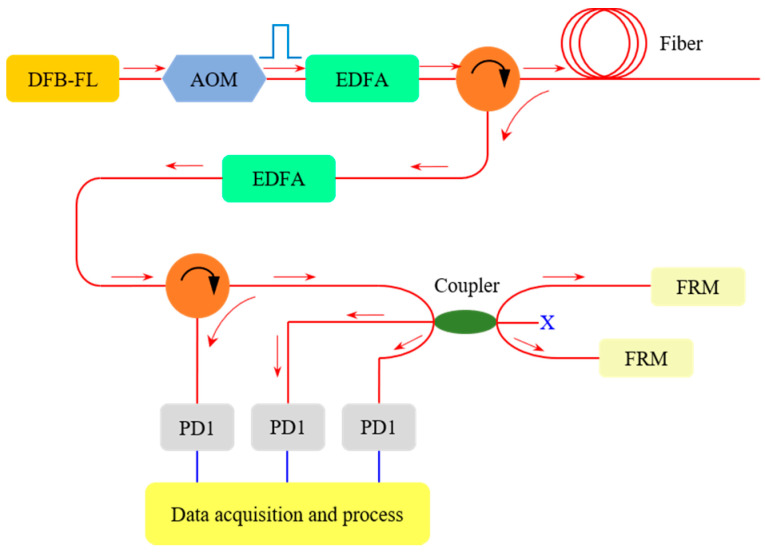
Experimental setup of the DAS system.

The setup of the DAS system is shown in Figure 4. The light source is a narrow linewidth distributed feedback fiber laser (DFB-FL) with a maximum output power of 30 mW and linewidth of 3 kHz. The CW light with a wavelength of 1550.12 nm is injected into an acoustic-optic modulator (AOM) to generate the pulses, whose width is 50 ns, and the repetition rate *R* is fixed at 20 kHz. An Erbium-doped fiber amplifier (A) is used to amplify the pulses, and the ASE noise is filtered by an optical fiber Bragg grating filter (F). Then, the amplified pulses are launched into a single mode detection fiber (Corning SMF-28e, Corning Incorporated, New York, NY, USA) by a circulator. The Rayleigh back-scattering is amplified (A) and filtered (F) again and then injected into a Michelson interferometer that consists of a circulator, a 3 × 3 coupler and two FRMs. The final interference signals’ outputtings are collected by three photodetectors (PD1~3) and then demodulated via the typical 3 × 3 passive demodulation algorithm with a 3 × 3 coupler [23], which contain distributed acoustic pressure responses that can be extracted.

## 3. Experimental Result and Discussion

First, we analyze the conclusions derived from the theory and simulate the characteristics of the pipeline fluid velocity and pipe wall pressure.

We use a three-dimensional model in the simulation, and the pipeline is simplified to a cylinder. We set a situation according to which the fluid in the pipeline flows from the lower pipe to the upper pipeline, so that the overall structure of the design is a U-shaped pipe model with 90° bends, the pipe material is iron, and the fluid material is water. The calculated Reynolds number shows that the fluid state in the pipe is turbulence, so the physical field chooses the turbulence *k-ω* model to study the steady state. Other specific pipe model parameters are shown in Table 1.

**Figure 5:**
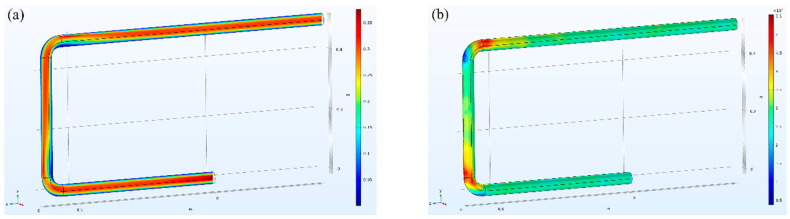
(**a**) Velocity field distribution. (**b**) Wall pressure distribution.

After the establishment of the model, the boundary conditions are set. The fully developed flow is selected at the entrance. The average velocity is set to 5 m/s. The pressure of 0 Pa is set at the exit, and the reflux is suppressed. Then, the steady-state solution is selected, which can be calculated directly. Figure 5 shows the distribution of fluid velocity in the pipe and the pressure of fluid on the pipe wall when the velocity is fixed.

**Figure 6:**
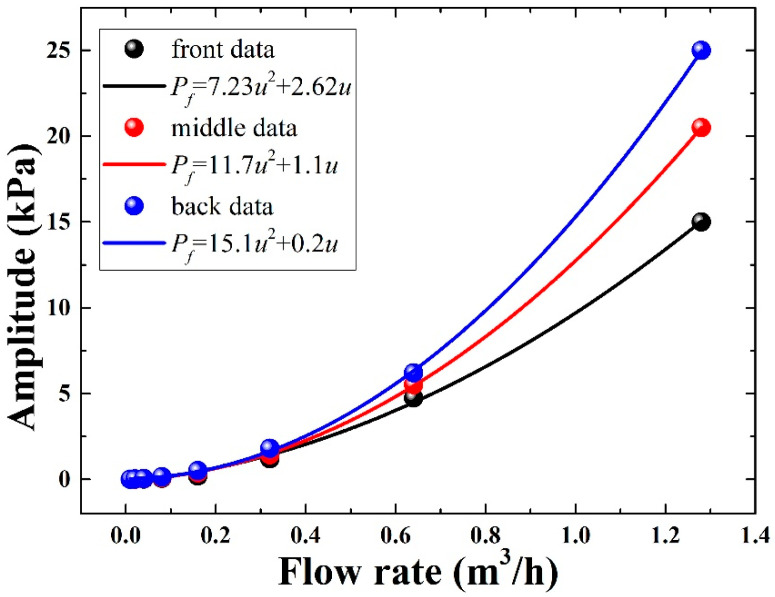
Relationship between flow rate and pipe wall pressure.

From the simulation results, it can be observed that there is a difference in the distribution of pressure between the front and back of the pipe wall near the bend. For the determined velocity, the stronger the impact on the pipe wall, the greater the energy obtained here, and the better the observed results will be in theory. Therefore, three three-dimensional cut-off points are added before, at and after the pipe bend, and the step size of the fluid velocity in the pipe is set, so that the fluid velocity in the pipe increases gradually from 0–1.5 m/s. The corresponding pressure is recorded for different flow values and fit with the pressure, as shown in Figure 6.

From the fitting results, we can see that there is a quadratic relationship between the flow rate u and the pipe wall pressure *p*. The change of the pressure can be obtained quantitatively through the detection of the phase difference of the DAS system, and the measurement of the flow can be realized.

**Figure 7:**
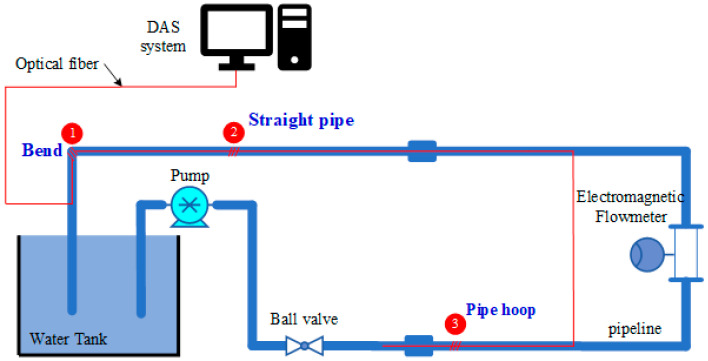
Experimental schematic diagram of flow detection based on the DAS system.

According to the simulation model, a flow test system is built. The schematic diagram of the experiment is shown in Figure 7. The fluid medium is water. The pipeline flow rate is measured by the DAS system under different fluid flow rates. Gradually increase the water flow in the pipe, record the phase changes collected in turn by the DAS system at different velocities, and record the actual flow measured by the electromagnetic flowmeter.

**Figure 8:**
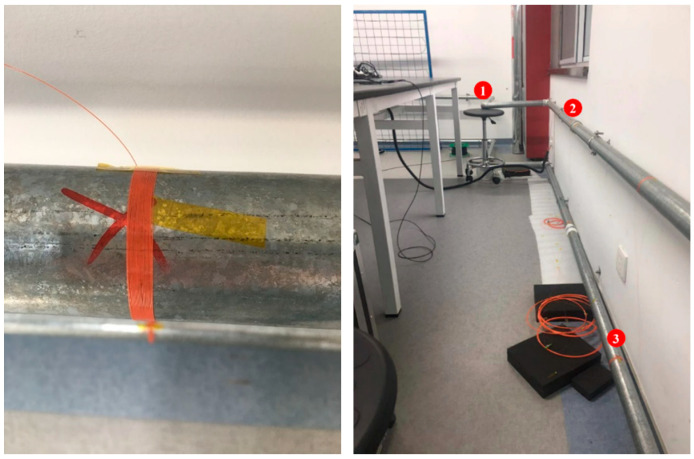
Experiment setup of the field test.

In addition, an optical fiber of about 3 m is wound and glue-fixed at the bend, the straight pipe and the pipe hoop of the water pipe, respectively, and there is about 70 m to 90 m of fiber between the two nearby fiber rings and also 40 m of fiber at the front end and tail end, respectively, to prevent the crosstalk of the signal (Figure 8), so that the total length of the sensing fiber is about 265 m. The flow velocity in the tube is continuously changed by pulling the valve, increasing from 0.73 m^3^/h to 2.48 m^3^/h. After each adjustment of the valve, the current electromagnetic flowmeter value is recorded for these three measurement points, which are used for subsequent experimental data processing.

**Figure 9:**
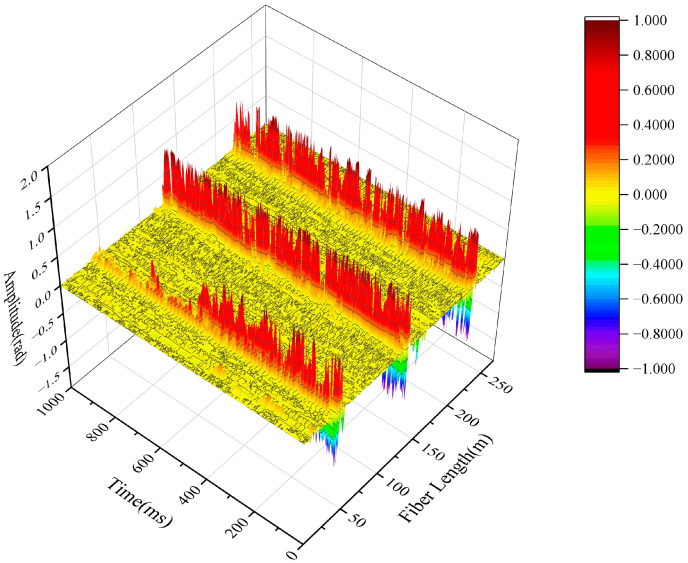
Pipeline winding fiber position.

Before the flow test, the optical fiber wound on the pipe needs to be located, the upper and lower pipes are tapped, and the location is analyzed after saving the data. The demodulated-phase diagram is shown in Figure 9 and can be seen on the whole section of optical fiber. The three sections of optical fiber wound on the pipe are clearly distinguished. The bend section is from 47 m to 50 m, and the straight pipe and pipe hoop sections are from 125 m to 128 m and from 222 m to 225 m.

**Figure 10:**
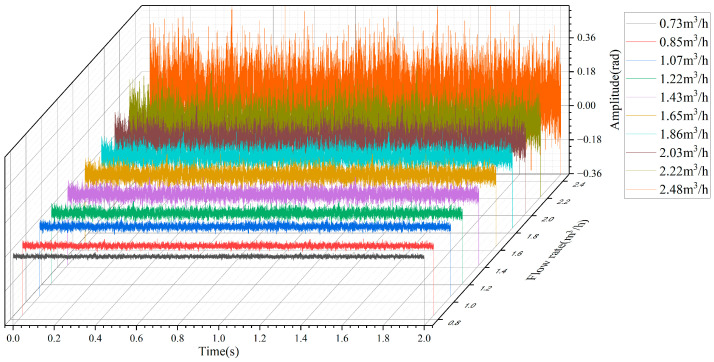
Time domain diagrams of demodulated phases in different flow rates.

**Figure 11:**
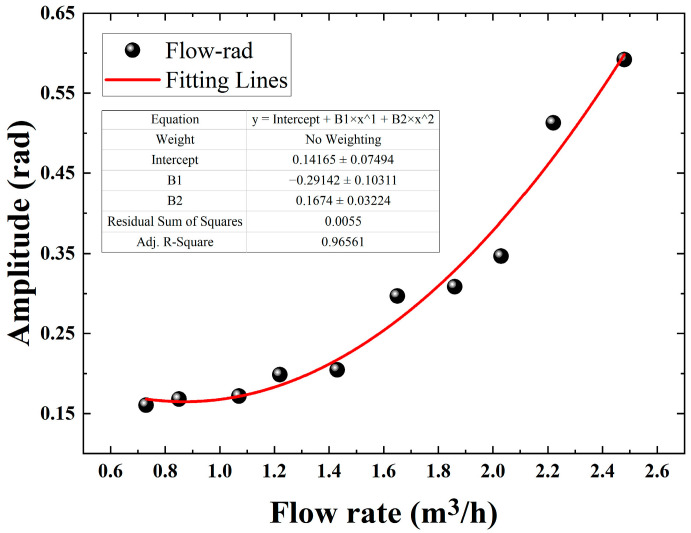
Experimental result of phase-flow rate relationship and the fitting curve.

After determining the position, we pick the phase data at the center of the bend section and change the flow value. In the experiment, 10 groups of flow rate values are measured, and the phase values from the sensing point corresponding to each group of flow values are recorded for 2 s. As shown in Figure 10, with the continuous increase of the flow rate, the fluctuation of demodulated phases in the time domain will also increase. The average Radian value of the three measurements at the same flow rate is taken as the DAS system demodulation value corresponding to each flow rate value, and the fitting result is shown in Figure 11.

**Figure 12:**
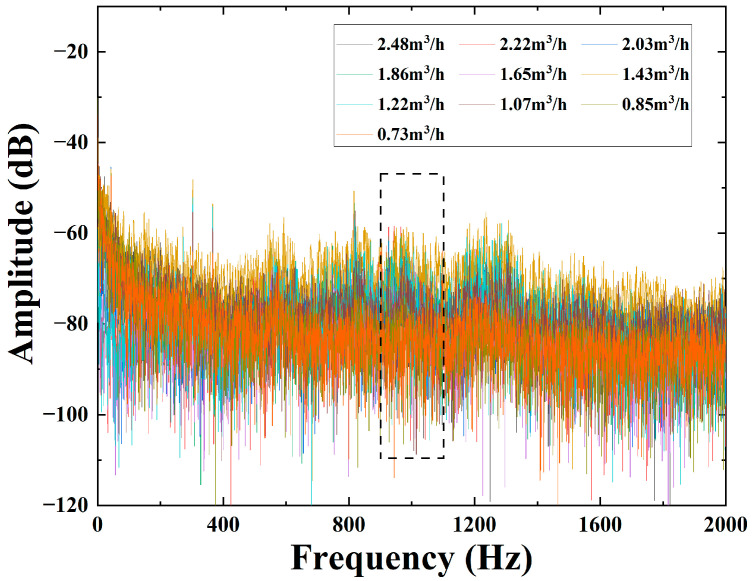
Frequency domain diagrams of demodulated phases in different flow rates.

**Figure 13:**
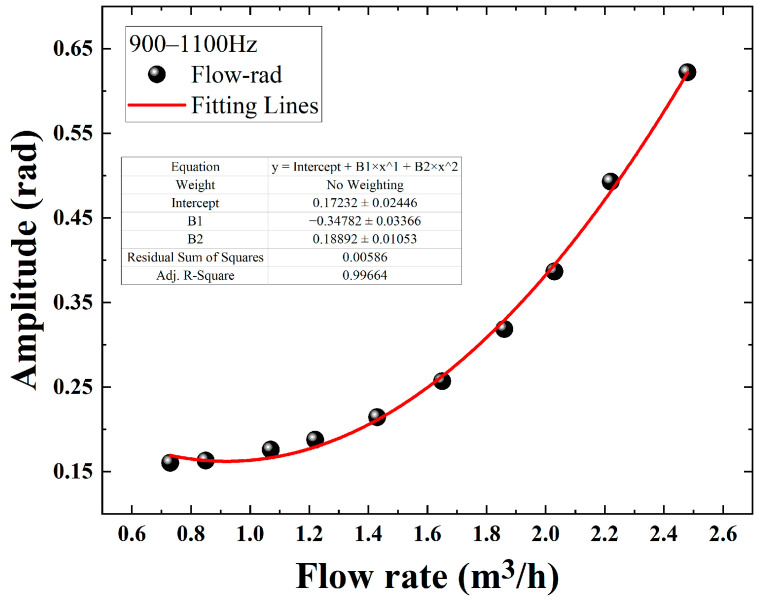
Phase-flow rate relationship in 900–1100 Hz region and the fitting curve.

One can see from Figure 10 that the demodulated phases via the DAS system have a quadratic relationship with the flow rate in the pipeline. The mathematical relationship obtained after the fitting follows a quadratic function with R^2^ = 0.965, which is consistent with the theory that the phase is proportional to the square of the velocity in the second chapter; but from the fitting results, there is some deviation in the corresponding demodulation phases at some flow rates. In order to get a better fitting effect, we calculate and analyze the frequency spectra of 10 groups’ data via the Fast Fourier transform (FFT). The unit dB in Figure 11 is for 10*log(rad). As shown in Figure 12, we can clearly see that the relationship between the flow and the demodulated phases from 900 Hz to 1100 Hz is more significant and sensitive, which may be caused by the local structure resonance of the test system. The average amplitude is calculated in this sensitive-response frequency region and then fitted with the flow value, and the fitting result is shown in Figure 13.

One can see from Figure 13 that the acoustic energy produced by the fluid to the pipe wall is mainly concentrated in the 900–1100 Hz frequency region, and the mathematical relationship obtained after fitting shows a good quadratic function with R^2^ = 0.997. Compared with the previous fitting result, R^2^ increases by 0.32, which indicates that the pressure energy of the fluid to the tube wall is more concentrated in a specific frequency region. At the same time, this is consistent with the theory that the phase is proportional to the square of the flow rate in the second chapter, and the fitting result is almost the same as the trend of the simulation, being only slightly different in value. The above experiments provide a theoretical basis for the practical application of flow detection. In addition, other peaks in Figure 11 are also automatically selected with a sliding frequency region window of 200 Hz and a step of 10 Hz from 0 Hz, calculated for fitting, and they all have a quadratic relationship with a lower R^2^.

**Figure 14:**
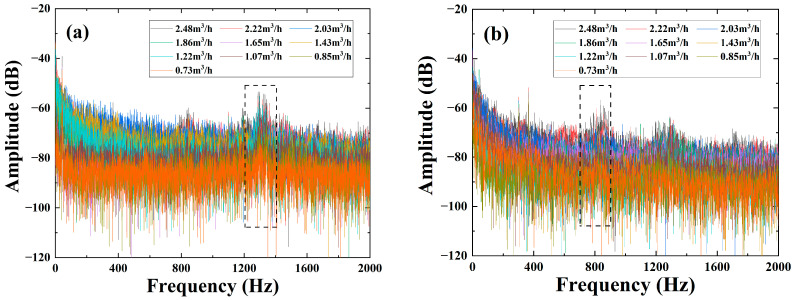
Frequency domain diagrams of the demodulated phases in different flow rates: (**a**) at the straight pipe; (**b**) at the pipe hoop.

**Figure 15:**
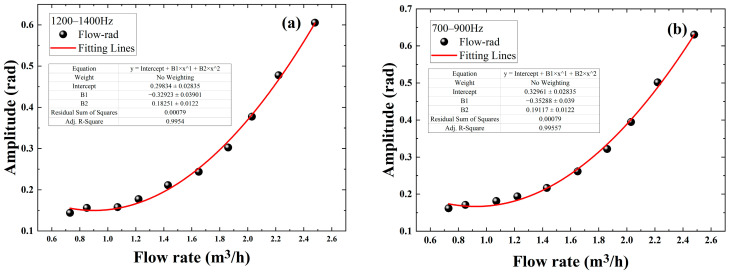
Phase-flow rate relationship in the sensitive-response frequency region and the fitting curve: (**a**) at the straight pipe; (**b**) at the pipe hoop.

After processing the data at the bend, the data measured at the other two positions of the straight pipe and the pipe hoop are also processed, the frequency domain diagram is shown in Figure 14, the sensitive-response frequency region is fitted, and the final fitting result is shown in Figure 15. The results clearly show that the phase-flow rate relationships at both the straight pipe and the pipe hoop follow the quadratic function, fitting well with the simulation. The difference in the sensitive-response frequency regions is mainly caused by the different positions measured. At the straight pipe it ranges from 1200 Hz to 1400 Hz, and at the pipe hoop it ranges from 700 Hz to 900 Hz. Further studies will focus on the choice of the sensitive-response frequency region and its mechanism on structures.

## 4. Conclusions

In this paper, we mainly propose a non-intrusive pipeline flow detection based on distributed fiber turbulent vibration sensing. The quantitative measurement of the flow rate is acquired theoretically though the amplitude of the demodulated phase changes from DAS based on the flow impact in the tube on the pipe wall. The experimental results show that the relationships between the flow rate and the demodulated phase changes in both a whole frequency region and in a sensitive-response frequency region fit the quadratic equation well, with a max R^2^ of 0.997, which is consistent with the theoretical simulation results. The detectable flow rate ranges from 0.73 m^3^/h to 2.48 m^3^/h. This work verifies the feasibility of the DAS system optical fiber flow measurement, which is of great significance for downhole flow detection, the optimization of oil recovery and the improvement of oil recovery.

## Figures and Tables

**Table 1:** Basic parameter setting of the pipeline model.

Name	Value	Description
D	40 [mm]	Pipe diameter
L_in_	600 [mm]	Entrance length
L_c_	500 [mm]	Connection length
L_out_	1000 [mm]	Exit length
R_c_	50 [mm]	Coil radius
R_hof_	965.35 [kg/m^3^]	Density
Muf	3.145 × 10^−4^ [Pa·s]	Dynamic viscosity
U_avg_	5 [m/s]	Average speed

## Data Availability

The data presented in this study are available on request from the corresponding author.
